# Recombinant Human Arresten and Canstatin Inhibit Angiogenic Behaviors of HUVECs via Inhibiting the PI3K/Akt Signaling Pathway

**DOI:** 10.3390/ijms23168995

**Published:** 2022-08-12

**Authors:** Lingyu Zhu, Zitao Guo, Ji Zhang, Yuliang Yang, Chunyu Liu, Liang Zhang, Zhenghua Gu, Youran Li, Zhongyang Ding, Guiyang Shi

**Affiliations:** 1 Engineering Research Center for Cereal Fermentation and Food Biomanufacturing, Jiangnan University, Wuxi 214122, China; 2 Jiangsu Provincial Engineering Research Center for Bioactive Product Processing, Jiangnan University, Wuxi 214122, China

**Keywords:** angiogenesis, arresten, canstatin, *Escherichia coli*

## Abstract

Angiogenetic inhibitors are crucial in tumor therapy, and endogenous angiogenesis inhibitors have attracted considerable attention due to their effectiveness, safety, and multi-targeting ability. Arresten and canstatin, which have anti-angiogenesis effects, are the c-terminal fragments of the α1 and α2 chains of type IV collagen, respectively. In this study, human arresten and canstatin were recombinantly expressed in *Escherichia coli* (*E. coli*), and their effects on the proliferation, migration and tube formation of human umbilical vein endothelial cells (HUVECs) were evaluated. Regarding the cell cycle distribution test and 5-ethynyl-2′-deoxyuridine (EdU) assays, arresten and canstatin could repress the proliferation of HUVECs at a range of concentrations. Transwell assay indicated that the migration of HUVECs was significantly decreased in the presence of arresten and canstatin, while tube formation assays suggested that the total tube length and junction number of HUVECs were significantly inhibited by these two proteins; moreover, they could also reduce the expression of vascular endothelial growth factor (VEGF) and the phosphorylation levels of PI3K and Akt, which indicated that the activation of the 3-kinase/serine/threonine-kinase (PI3K/Akt) signaling pathway was inhibited. These findings may have important implications for the soluble recombinant expression of human arresten and canstatin, and for the related therapy of cancer.

## 1. Introduction

Angiogenesis, the process of promoting new blood vessels from existing ones, is necessary for wound healing, inflammation, and embryonic development [1]. In 1971, Folkman et al. first proposed a hypothesis that tumor growth is dependent on angiogenesis [2]. After that, anti-angiogenesis became one of the most important strategies for cancer treatment [3]. Cancer is one of the world’s most deadly diseases, taking the lives of millions of people each year. Currently, the discovery and development of novel anti-cancer drugs is a research hotspot. Numerous angiogenetic inhibitors were found and developed as anti-cancer drugs, such as monoclonal antibodies, small-molecule drugs, and microRNAs and protein fragments, among which endogenous inhibitors have received more attention due to their low toxicity, minimal danger of drug resistance, high tolerance, and higher probability of specifically preventing abnormal neovascularization without disrupting the normal vasculature. Basement membranes (BMs) are nanoscale, delicate, and flexible sheets of extracellular matrix that are widely distributed in metazoan tissues, and serve as linings or dividers in organisms [4]. Recently, BMs have been identified as vital cellular regulators, rather than only an architectural support. It has been reported that BMs constitute a necessary component of vascular structure and function, and several BM components are involved in the regulation of tumor angiogenesis [5,6].

Angiogenesis and tumor growth have been demonstrated to be inhibited by the non-collagenous (NC1) structural domain of several type IV collagen α chains [7,8,9]. Previous studies have demonstrated that type IV collagen is crucial for endothelial cell proliferation and cellular activity [10]. The α1 and α2 chains of type IV collagen are abundantly produced in the basement membranes throughout the body, including blood vessels, glomerulus, bronchioles, and glands. Arresten is an endogenous angiogenesis inhibitor produced from the type IV collagen α1 chain’s NC1 structural domain [11]. Canstatin is the c-terminal fragment of the α2 chain of type IV collagen, which was first identified as endogenous anti-angiogenesis and anti-tumor factor by Kamphaus et al. in 2000, with a molecular weight of 24 kDa [9]. Recombinant arresten and canstatin have been produced by several types of expression systems, such as insect cells, mammalian cells, and yeast systems [12,13,14]. The eukaryotic expression system, however, is more expensive and time-consuming than the *Escherichia coli* (*E. coli*) approach. The *E. coli* expression system has the advantages of a high expression level, the capability of rapid growth, a low-cost and a well-characterized genetic background, and is widely used in large-scale protein production. Several studies have used *E. coli* hosts for the expression of human-derived arresten and canstatin, but have obtained recombinant proteins in the form of an inclusion body [1,15]. Compared to inclusion body proteins, soluble proteins have an intact spatial structure and improved biological activity. Therefore, it is necessary to express recombinant human arresten and canstatin in soluble form in *E. coli*.

In this study, we obtained soluble recombinant human arresten and canstatin using *E. coli* as the expression host. The effects of arresten and canstatin on human umbilical vein endothelial cells (HUVECs) on proliferation, migration, and tube formation were investigated. The findings suggest that recombinant human arresten and canstatin may bind to cell surface integrins and inhibit HUVEC proliferation, migration, and tube formation by blocking the activation of the phosphoinositide 3-kinase/serine/threonine-kinase (PI3K/Akt) signaling pathway. This study provides a novel strategy for the acquisition of recombinant human arresten and canstatin, and contributes to the research of endogenous angiogenesis inhibitors in tumor therapy.

## 2. Results

### 2.1. Construction, Expression, and Identification of Recombinant Human Arresten and Canstatin

**Figure 1:**
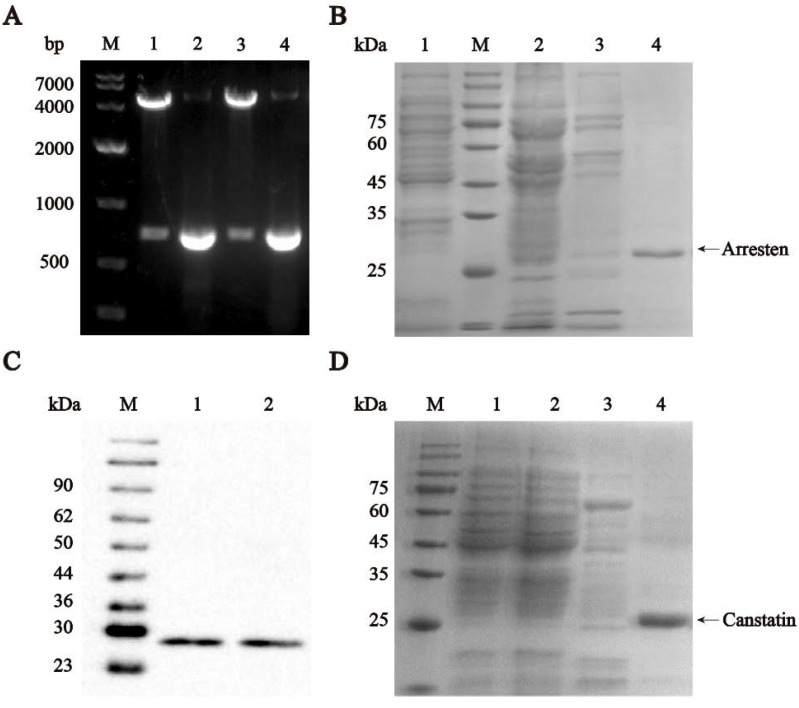
Construction of recombinant plasmids pRham^TM^-arresten and pRham^TM^-canstatin, and protein expression and identification. (**A**) Nucleic acid electrophoresis of re-combinant plasmid. M, DNA marker; Lane 1, pRham^TM^-arresten plasmid digestion product; Lane 2, PCR product of arresten; Lane 3, pRham^TM^-canstatin plasmid digestion product; Lane 4, PCR product of canstatin. (**B**) SDS-PAGE detection of the expression of recombinant arresten. M, protein marker; Lane 1, flow through; Lane 2, total protein of recombinant arresten; Lane 3, flow through. Lane 4, purified recombinant arresten. (**C**) Western blot identification of recombinant arresten and canstatin. M, Western blot molecular weight marker; Lane 1, recombinant arresten; Lane 2, recombinant canstatin. (**D**) SDS-PAGE detection of the expression of recombinant canstatin. M, protein marker; Lane 1, total protein of recombinant canstatin; Lane 2 and 3, flow through; Lane 4, purified recombinant canstatin. The protein bands for arresten and canstatin are indicated by arrows.

The cDNA of arresten and canstatin were reconstructed in pRham^TM^ plasmids. The SDS-PAGE results indicated that the purified proteins had significant bands at approximately 28 kDa, consistent with the theoretical molecular weights of arresten and canstatin, respectively (Figure 1B,D). Moreover, they were further confirmed by Western blot assay using anti-his-tag antibodies (Figure 1C).

### 2.2. Effects of Recombinant Human Arresten and Canstatin on Proliferation and Migration of HUVECs

**Figure 2:**
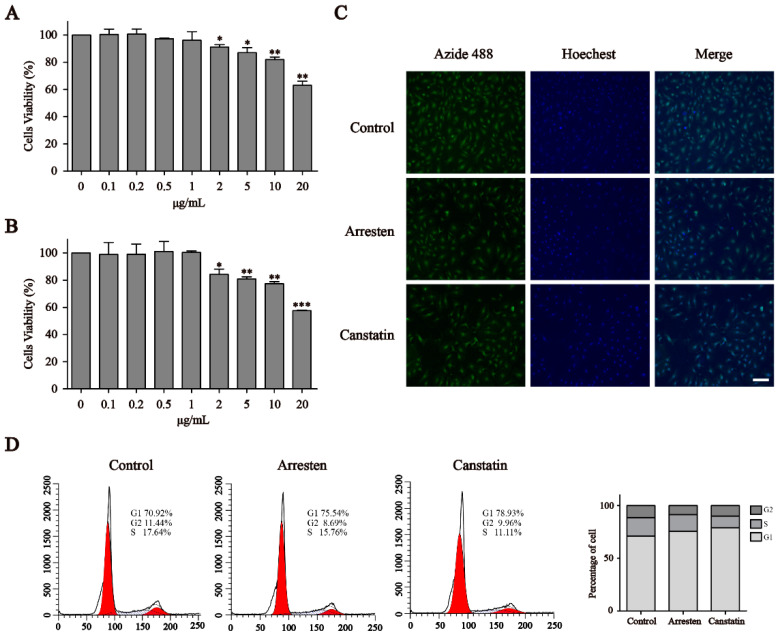
Effect of human arresten and canstatin on the proliferation of HUVECs. (**A**,**B**) The proliferation effects of recombinant human arresten and canstatin on HUVECs according to the CCK-8 assay. (**C**) Determination of cell proliferation using EdU. (**D**) Effects of recombinant human arresten and canstatin on the cell cycle distribution of HUVECs. Data are expressed as the mean ± standard deviation of triplicate experiments. Scale bars, 100 μm. * *p* < 0.05, ** *p* < 0.01, and *** *p* < 0.001 indicate a significant difference compared to the control group.

Cell Counting Kit-8 (CCK-8) assays indicated that arresten and canstatin could significantly inhibit the proliferation of HUVECs over 2 μg/mL. (Figure 2A,B). However, both arresten and canstatin had no effect below 2 μg/mL. Within the range of certain concentration, inhibition levels of arresten and canstatin on HUVECs were dose dependent. Given that arresten and canstatin could inhibit cell proliferation, the cell division was further investigated through EdU staining. As shown in Figure 2C, the frequencies of the green 5-ethynyl-20-deoxyuridine (EdU) fluorescent signal significantly decreased in the HUVECs treated with 20 μg/mL arresten and canstatin proteins, respectively.

To further clarify the inhibitory effects of arresten and canstatin on endothelial cell proliferation, the alteration of the cell cycle of HUVECs in both treatment groups was investigated by employing flow cytometry. The results indicated that the proportion of cells in G1 phase was increased and the percentages of cells in S and G2 phases were reduced in the arresten and canstatin groups, compared with the control group (Figure 2D). Recombinant arresten and canstatin could affect HUVEC proliferation through G1 arrest.

**Figure 3:**
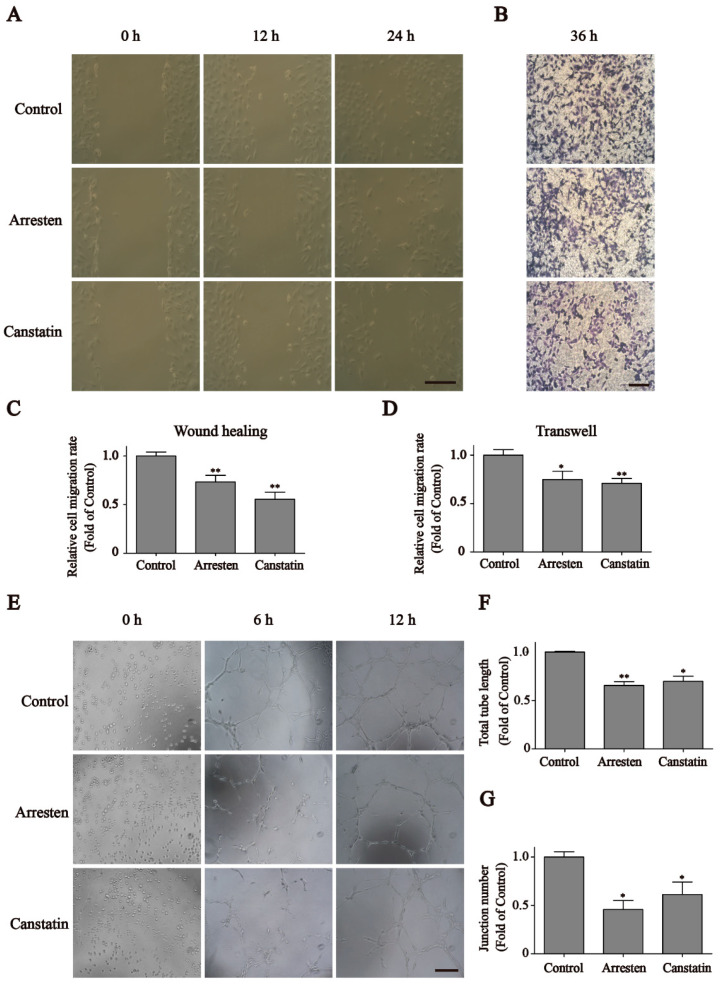
The influence of recombinant arresten and canstatin on HUVEC migration. (**A**) Assessment of the migration ability using wound healing test. (**B**) The Transwell assay indicates that arresten and canstatin significantly inhibited the HUVECs migration and shows the images obtained with a microscope at 100× *g* magnification. (**C**,**D**) The result of wound healing (24 h) and migration (36 h) is represented as the relative cell migration rate. (**E**) Tube formation assay of HUVECs. (**F**,**G**) total length and junction number of HUVEC tube formation after 12 h of recombinant arresten and canstatin treatment. Cells were treated with 20 μg/mL of arresten and canstatin proteins for 24 h, respectively. Scale bars, 200 μm. Data are expressed as the mean ± standard deviation of triplicate experiments. * *p* < 0.05 and ** *p* < 0.01 indicate a significant difference compared to the control.

To explore the effects of recombinant human arresten and canstatin on the migration of HUVECs, wound healing and Transwell assays were performed. The results revealed that the HUVECs migration rates were significantly reduced after treatment with a 20 μg/mL concentration of arresten and canstatin compared to the control group (Figure 3A). After 24 h of treatment, the migration rates in groups arresten and canstatin were 73.4% and 55.5% of those of the control group, respectively (Figure 3C). In order to confirm the effect of arresten and canstatin on cell migration, we performed a Transwell assay. As shown in Figure 3B, HUVECs treated with recombinant proteins for 36 h exhibited a significantly lower migration rate than the control group. The migration rate of the canstatin group (71.0%) was slightly lower than arresten group (74.9%), and these results were consistent with those of the wound healing assay (Figure 3D).

### 2.3. Effects of Recombinant Human Arresten and Canstatin on Tube Formation of HUVECs

Based on the results of wound healing and Transwell assays, we further validated the inhibition effects of recombinant arresten and canstatin on HUVECs’ migration by employing a tube formation assay. Figure 3E indicated the total length and junction number of HUVEC tube formation among the three groups. After incubation for 12 h, the total length and junction number in groups arresten (45.8% and 61.3%, respectively) and canstatin (69.8% and 61.3%, respectively) were significantly lower than those of the control group (Figure 3F,G). These results revealed that recombinant human arresten and canstatin could inhibit the tube formation capacity of HUVECs.

### 2.4. Effects of Recombinant Arresten and Canstatin on PI3K/Akt Signaling Pathway in HUVECs

**Figure 4:**
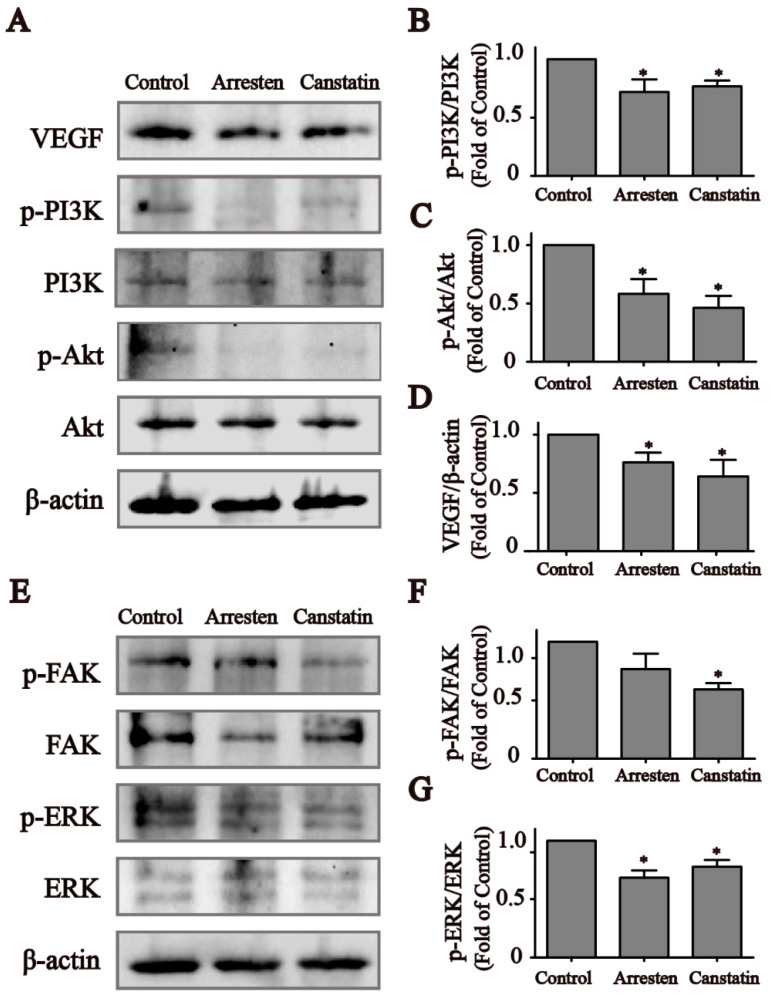

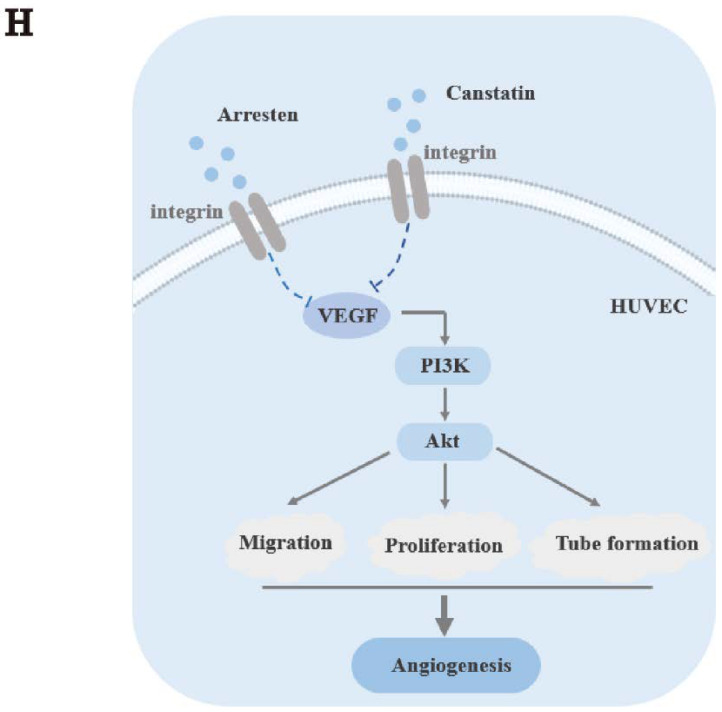
Effect of recombinant human arresten and canstatin on PI3K/Akt signaling pathway. (**A**) Western blot analysis of VEGF, p-PI3K, PI3K, p-Akt, and Akt proteins in different groups. (**B**–**D**) Protein expression levels of VEGF, p-PI3K, and p-Akt. (**E**) Western blot analysis of p-FAK, FAK, p-ERK, and ERK proteins in different groups. (**F**,**G**) Protein expression levels of p-FAK and p-ERK. (**H**) Schematic diagram of the mechanism by which recombinant human arresten and canstatin could bind to cell surface integrins and inhibit HUVEC proliferation, migration, and tube formation. Recombinant arresten and canstatin reduce VEGF expression and inhibit HUVEC proliferation, migration, and tube formation by suppressing activation of the PI3K/Akt pathway, thereby inhibiting angiogenesis. Data are expressed as the mean ± standard deviation of triplicate experiments. * *p* < 0.05 indicates a significant difference compared to the control.

As Focal adhesion kinase (FAK) and extracellular regulated protein kinases (ERK) play a pivotal role in integrin-mediated adhesion, we investigated the effect of recombinant arresten and canstatin on the phosphorylation of these two kinases. The expression levels of p-FAK and p-ERK in group arresten and canstatin was decreased (Figure 4F,G). PI3K/Akt represents a major mechanism to regulate cell survival and proliferation. To elucidate whether recombinant arresten and canstatin affect HUVEC proliferation through the PI3K/Akt pathway, we measured the expression levels of relevant markers (Figure 4A). As shown in Figure 4B–D, the expression levels of vascular endothelial growth factor (VEGF), p-PI3K, and p-Akt in the arresten and canstatin groups were significantly decreased compared with the control group, whereas there was no significant difference in the expression level of Akt. The results indicated that the recombinant human arresten and canstatin could bind to cell surface integrins and inhibited the activation of the PI3K/Akt pathway by reducing the expression of VEGF (Figure 4H).

## 3. Discussion

Angiogenesis is a complex physiological process regulated by cell release factors that coordinate the activity of endothelial and smooth muscle cells, with the goal of repairing damaged blood arteries [16]. To preserve physiological homeostasis, angiogenesis needs to be regulated by a balance of stimulants and inhibitors [17]. Pathological angiogenesis can result from a disruption in the balance of regulatory components, which can lead to disorders including heart disease, arthritis, and cancer [18]. The vascular system is the greatest physiological cellular network shared by all organs, and blood vessels are subject to ageing and diminished function as with other organ systems [19]. The vascular system regulates tissue function by providing oxygen, nutrients, and angiocrine signals [20]. Ageing is a risk factor for vascular disease, and vascular ageing has a greater impact on organ function. Age-associated alterations of the hormone-secreting endocrine system lead to organ dysfunction and disease states [21]. Studies have shown that ensuring adequate VEGF signaling by compensating for increased circulating VEGF levels can prevent age-related capillary loss and improve organ function in mice [22]. Numerous researchers have investigated the regulatory systems that promote or inhibit angiogenesis, notably in the context of tumor growth and metastasis [23]. Tumor vascularization is primarily through the release of strong vascular growth factors, such as VEGF and basic fibroblast growth factor (bFGF), which germinate from pre-existing vessels in the vicinity of the tumor. Currently, the most familiar techniques of VEGF suppression include blocking the VEGF receptor or ligand by neutralizing antibodies and inhibiting the receptor tyrosine kinase. At present, the most popular anti-angiogenic medications include monoclonal antibodies, tryosine kinase inhibitors, and mTOR inhibitors [24]. Although these anti-angiogenic medications have a part to play in the treatment of cancer patients, their clinical application is limited by their minimal efficacy, drug resistance, and potential safety issues [25]. As a consequence, it is now necessary to design innovation inhibitors that are low resistance, safe, and multi-targeted. Currently, endogenous angiogenesis inhibitors, which are mainly proteins or fragments formed in vivo, are widely used due to their non-toxicity for normal cells and lower drug resistance [26]. In recent years, basement membranes, particularly endogenous angiogenesis collagen-derived inhibitors, have received a lot of attention. The complex structure, consisting of multiple glycoproteins, proteoglycans, and hyaluronic acid, is called the extracellular matrix (ECM). Endogenous matrix-derived angiogenesis inhibitors are bioactive protein fragments derived from ECM proteins, including type XVIII and type IV collagens. Type IV collagen, the primary component of BMs, is a complex network composed of triple helix collagen molecules. The C-terminal domain of type IV collagen plays a major role in chain assembly and has been referred to as the NC1 domain. Arresten and canstatin are two inhibitors of angiogenesis derived from precursor human collagen molecules known as α1 and α2 chains of type IV collagen.

Due to the specificity of the sample source, human-derived inhibitors of angiogenesis are usually produced by heterologous expression. Currently, the exogenous recombinant expression of endogenous matrix-derived angiogenesis is achievable using HEK-293 cells, Drosophila S2 cells, *Pichia pastoris*, and *E. coli* BL21 [9,13,27,28]. In comparison to microbial expression systems, insect and mammalian cell expression methods are costly, technically complex, and have low yields. When exogenous genes are expressed in yeast, inadequate signal peptide processing, poor protein homogeneity and polymer formation would increase difficulties for large-scale production [29]. *E. coli* is commonly utilized as a host for the expression of exogenous proteins due to its clear genetic background, easy operation, fast growth cycle, and low cost. At present, several studies utilizing *E. coli* as the host employ inclusion body or tag fusion expression to produce recombinant endogenous matrix-derived angiogenesis inhibitors [30,31]. However, these production strategies waste more energy on the separation and purification of the products, which would significantly increase the cost of production. In this study, soluble recombinant human arresten and canstatin were obtained through heterologous expression using *E. coli* as the host (Figure 1), and the angiogenesis inhibition of the recombinant proteins for HUVECs was investigated.

First, the CCK-8 assay indicated that recombinant human arresten and canstatin could inhibit the proliferation of HUVECs in a dose-dependent manner (Figure 2A,B). Then, we used a 20 μg/mL concentration of arresten and canstatin to carry out subsequent experiments. The EdU assay also showed reduced cell proliferation in the arresten and canstatin groups compared with the control group. After treatment with recombinant proteins, cell cycle analysis revealed a decrease in S phase cells and an increase in G1 phase (Figure 2D). These results showed that arresten and canstatin could effectively inhibit the transition of cells from G1-Phase to S-phase. Wound healing and Transwell assays showed that recombinant human arresten and canstatin could inhibit the migration of HUVECs, and the migration rate of the canstatin group was lower than that of the arresten group (Figure 3C,D). A classical in vivo model for evaluating angiogenesis is tube formation [32]. In this study, recombinant human arresten and canstatin exhibited an inhibitory effect on HUVEC tube formation, as indicated by the considerable reduction in the total length and junction number in the arresten and canstatin groups. In view of this, these results indicated that the recombinant arresten and canstatin obtained in this study possess the anti-angiogenesis effects on HUVECs. Arresten and canstatin have been shown to bind to integrins and exert anti-angiogenic effects in endothelial cells [33,34]. Integrins are heterodimeric cell surface receptors consisting of a combination of different a and b subunits, the abnormal expression of different integrin pairs is frequently related to the occurrence of various pathological conditions [35]. FAK is a nonreceptor tryosine kinase whose phosphorylation activity is regulated by integrin-medicated cell adhesion [36]. Several studies have suggested that integrin-induced signaling may regulate cell proliferation and survival through ERK [37].The PI3K/Akt pathway is broadly distributed in biological cells and is essential for numerous physiological and pathological processes, participating in the regulation of proliferation, cell cycle, apoptosis, and migration [38] The PI3K/Akt pathway is broadly distributed in biological cells and is essential for numerous physiological and pathological processes. The major function of PI3K, which belongs to the phosphatidylinositol family, is to activate other substances and produce second messengers [39]. Subsequently, a chain reaction would be set off by downstream molecules that are activated by the second messengers. Akt, which is also known as protein kinase B, is a serine/threonine specific protein kinase and one of the downstream targets of PI3K. It is involved in a variety of processes, including proliferation, cell growth, transcriptional regulation, and protein synthesis [40]. VEGF, an essential growth factor for endothelial cells that promotes the proliferation, differentiation, and survival of endothelial cells, is primarily responsible for angiogenesis [41]. Additionally, during angiogenesis, the increase in VEGF is closely related to the activation of the PI3K/Akt signaling pathway [42]. Thus, VEGF silencing can inhibit cell proliferation and promote apoptosis by suppressing the PI3K/Akt signaling pathway [43]. The expression levels of p-FAK and p-ERK indicated that arresten and canstatin could bind to integrins on the cell surface. To determine whether recombinant human arresten and canstatin affect HUVEC proliferation and migration through the PI3K/Akt signaling pathway, we investigated the expression levels of VEGF, p-PI3K, and p-Akt. Our findings implied that treatment with recombinant human arresten and canstatin could inhibit the PI3K/Akt signaling pathway in HUVECs by down-regulating the expressions of VEGF, p-PI3K, and p-Akt. Endostatin is the C-terminal fragment of type XVIII collagen, and is able to target a wider range of signaling molecules and receptors than anti-angiogenic therapeutics, such as tyrosine kinase inhibitors and monoclonal antibodies. Meanwhile, Endogenous matrix-derived angiogenesis has been certified to induce less drug resistance and fewer side effects [26]. Endostatin inhibits endothelial cell survival by regulating the PI3K/Akt signaling pathway, with similarities to our findings [44].

In summary, our study achieved the soluble heterologous expression of human arresten and canstatin, and demonstrated that recombinant human arresten and canstatin could bind to cell surface integrins and inhibit the proliferation, migration, and tube formation of HUVECs by inhibiting the activation of the PI3K/Akt signaling pathway.

## 4. Materials and Methods

### 4.1. Chemical Reagents

The *E. coli* BL21 (DE3) strain was used in this research as a host for the expression of recombinant arresten and canstatin. The DNA ligase, Taq DNA polymerase, and restriction endonucleases were obtained by Takara. The BCA Protein Assay kit was obtained from Solarbio Biotechnology (Beijing, China). Matrigel was purchased from ABW Biotechnology company (Shanghai, China). The EdU cell proliferation kit, cell cycle, and apoptosis analysis kit and CCK-8, and anti-VEGF (AF0312), anti-Akt (AF0045), anti-p-Akt (AF1546), anti-PI3K (AF7742), anti-p-PI3K (AF5905), anti-FAK (AF1108), anti-p-FAK (AF1906), anti-ERK (AF1051), and anti-p-ERK (AF1891) antibodies were purchased from Beyotime Biotechnology (Jiangsu, China). Anti-6 × His tag (ab213204) was purchased from Abcam corporation (Cambridge, UK). Anti-β-actin (sc-47778) was obtained from Santa Cruz Biotechnology (Dallas, TX, USA). The luminol reagent, albumin bovine V, anti-mouse, and anti-rabbit secondary antibodies were purchased from Lablead Biotechnology (Beijing, China). Other chemicals and reagents were of analytical grade.

### 4.2. Construction of Expression Plasmids

The plasmids pUC57-arresten and pUC57-canstatin were constructed by Tianlin Biological Company. The plasmids pUC57-arresten and pUC57-canstatin were used as a template to obtain the human *arresten* (ABE73157.1) and *canstatin* (AAF72631.1) genes through PCR. Arresten and canstatin fragments were obtained using arr-f/r and can-f/r, and cyclizing to plasmid, respectively (Table 1). The amplified PCR fragments of arresten and canstatin were cloned into NcoI and *Hind*III sites of pRham^TM^ to obtain the expression plasmids pRham^TM^-arresten and pRham^TM^-canstatin. Then, the recombinant plasmids were transformed into *E. coli* BL21(DE3), and positive transformants were deposited on Luria-Bertani (LB) agar plates supplemented with ampicillin (100 μg/mL). Subsequently, the inserted fragments were verified using agarose gel electrophoresis and DNA sequencing.

### 4.3. Recombinant Expression and Purification of Arresten and Canstatin

*E. coli* BL21(DE3) was used to express the proteins, and the medium used for the cultivation of *E. coli* was the LB medium (10 g/L NaCl, 10 g/L tryptone and 5 g/L yeast extract). The expression strains were inoculated in the LB medium at 37 °C and incubated at 200 rpm for 12 h. Then, 5% (*v*/*v*) of the culture medium was inoculated into fresh LB medium. After that, rhamnose (10 g/L) was added into the medium to induce protein expression when the optical density at 600 nm (OD_600_) was 0.8. The culture was induced for 24 h at 16 °C. Then, the cells were harvested and resuspended in the buffer (50 mM Tris, 500 mM NaCl; pH 8.0), and lysed by sonication. The his-tagged recombinant arresten and canstatin were purified using the Ni-NTA column (Qianchun, China) according to the manufacturer’s instructions. Purified recombinant arresten and canstatin dissolved in phosphate buffered saline (PBS) solution. The purified proteins were analyzed by 12% SDS-polyacrylamide gel electrophoresis (SDS-PAGE).

### 4.4. Cell Culture

The human umbilical vein endothelial cells (HUVECs) were obtained from the China Center for Type Culture Collection (Wuhan, China). HUVECs were cultured in endothelial cell medium (ScienCell, Carlsbad, CA, USA), supplemented with 1% penicillin/ streptomycin, 1% endothelial cell, and 5% FBS, and incubated at 37 °C in a humidified 5% CO_2_ atmosphere. The medium was changed every two days and the cells were passaged using 0.25% trypsin when they reached 80% to 90% cell fusion.

### 4.5. CCK-8 Assay

CCK-8 was used to evaluate the proliferation of HUVECs. HUVECs (2 × 10^3^) were seeded in 96-well plates. After 12 h of incubation, cells were treated with recombinant arresten and canstatin at different concentrations (0.1, 0.2, 0.5, 1, 2, 5, 10, and 20 μg/mL) for 24 h. An amount of 10 μL CCK-8 reagent was added to each cell, followed by incubation for 2 h at 37 °C. Then, the absorbance of each sample was measured at 450 nm using a spectrophotometer.

### 4.6. EdU Assay

EdU incorporation assay was evaluated with the EdU assay kit following the manufacturer instructions. Briefly, HUVECs were seeded in a 24-well plate and treated with 20 μg/mL doses of arresten and canstatin for 24 h when they reached the desired density (about 70%). Then, each sample was incubated with 10 μM EdU for 2 h. Next, the medium was removed and the cells were fixed for 15 min at room temperature with 4% paraformaldehyde. Subsequently, the cells were washed three times in PBS and permeabilized for 15 min at room temperature with 0.3% TritonX-100. Then, each sample was rinsed twice in PBS solution containing 3% BSA. Finally, the cells were reacted with a click addictive solution containing Azide 488 fluorescent dye in the dark for 30 min at room temperature, followed by incubation with Hoechst 33342 (dark, 10 min, room temperature). The images were obtained using Leica inverted microscope and the data were analyzed using Image J.

### 4.7. Flow Cytometry Analysis

Flow cytometry was performed to detect the HUVEC cell cycle. HUVECs were plated in 6 cm plates for 12 h, and then treated with a 20 μg/mL concentration of recombinant arresten and canstatin, respectively. The treated cells were incubated at 37 °C in the 5% CO_2_ incubator for 24 h. After that, they were rinsed with PBS and digested with trypsin. Cells were fixed with precooled 70% ethanol for 24 h and 0.5 mL Propidium Iodide (PI) solution was added to stain the cells for 30 min at 37 °C in the dark. For each sample, 2 × 10^4^ events were analyzed using a flow cytometer.

### 4.8. Wound Healing Assay

Cell migration was assessed using a wound healing assay. HUVECs were cultured in a 6-well plate to form a tight cell monolayer. Then, the cell monolayer was scratched by a plastic tip to create a regular and defined wound within the cell monolayer. The cells were washed three times with PBS to remove floating cells and debris, and then incubated for 24 h at 37 °C with serum-free DMEM or serum-free DMEM containing 20 μg/mL recombinant protein. The wounds of cell scratches were photographed at 0 h, 12 h, and 24 h. The migration rate of the HUVECs was calculated using Image J.

### 4.9. Transwell Assay

Transwell chambers (Corning, New York, NY, USA) with pores of 8 μm were used for cell migration. The HUVECs (4 × 10^4^ per well) were seeded in the upper compartment of a 24-well Transwell plate containing serum-free medium with 20 μg/mL of recombinant protein. Meanwhile, 700 μL endothelial cell medium containing 5% FBS and 1% ECGS was added to the bottom compartment. After 36 h incubation, the medium was removed and the chamber was washed three times with PBS. The migrated cells were fixed with 4% formaldehyde for 20 min and stained with 0.1% crystal violet for 30 min in the dark. The cells were observed and counted under a microscope (Leica, DMI1).

### 4.10. Tube Formation Assay

The thawed Matrigel matrix (50 µL) was added to each well in 96-well plates and incubated at 37 °C for 30 min to polymerize. Then, the HUVECs (1 × 10^4^ per well) cells were seeded in each well, and treated with a 20 μg/mL concentration of recombinant arresten and canstatin. After incubation for 12 h at 37 °C, tube formation was observed using a microscope.

### 4.11. Western Blot Assay

HUVECs were treated with arresten and canstatin for 24 h at a concentration of 20 μg/mL, respectively. Then, the total cellular protein was extracted and the concentration was determined using the BCA protein assay kit according to the manufacturer’s instruction. The same amounts of protein (15 μg) were mixed in a 4:1 ratio with 5 × SDS-PAGE loading buffer. The 10% SDS-PAGE was employed to separate the proteins, which were subsequently wet transferred onto a PVDF membrane for 80 min at 350 mA. After that, the membranes were blocked with 5% BSA in TBST (10 mM Tris-HCl, 150 mM NaCl, 0.1% tween 20) and then incubated with primary antibodies overnight at 4 °C. Next, the membranes were rinsed with TBST and incubated for 1 h at room temperature with secondary antibody. Immunoreactive signals were observed using luminol reagent after washing with TBST.

### 4.12. Statistical Analysis

The SPSS commercial statistical tool (version 21.0, Chicago, IL, USA) was used to perform the statistical calculations. Data are presented as the mean ± standard deviation. A one-way analysis of variance was used to assess the data, and a *p*-value below 0.05 was declared statistically significant. GraphPad Prism was used to create the figures (version 7.0, La Jolla, CA, USA).

## Figures and Tables

**Table 1:** Primers used to construct plasmids.

Primers	Sequences (5′-3′)
arr-f	CATGCCATGGATGTCAGTTGATCACGGCTTC
arr-r	GGGAAGCTTGGTACGACGCATGCAAACC
can-f	CATGCCATGGATGGTGAGCATCGGCTACCT
can-r	GGGAAGCTTCAGGTTTTTCATGCAAAC
